# Newborn screening of glucose-6-phosphate dehydrogenase deficiency in Guangxi, China: determination of optimal cutoff value to identify heterozygous female neonates

**DOI:** 10.1038/s41598-017-17667-6

**Published:** 2018-01-16

**Authors:** Chunyun Fu, Shiyu Luo, Qifei Li, Bobo Xie, Qi Yang, Guoxing Geng, Caijuan Lin, Jiasun Su, Yue Zhang, Jin Wang, Zailong Qin, Jingsi Luo, Shaoke Chen, Xin Fan

**Affiliations:** 1 Department of Genetic Metabolism, Maternal and Child Health Hospital of Guangxi Zhuang Autonomous Region, Nanning, 530003 China; 2 Research Center for Guangxi Birth Defects Control and Prevention, Nanning, 530003 China; 3 Guangxi Huayin Medical Laboratory Center, Nanning, 530012 China; 4 Department of Pediatrics, Maternal and Child Health Hospital of Guangxi Zhuang Autonomous Region, Nanning, 530003 China

## Abstract

The aim of this study is to assess the disease incidence and mutation spectrum of glucose-6-phosphate dehydrogenase (G6PD) deficiency in Guangxi, China, and to determine an optimal cutoff value to identify heterozygous female neonates. A total of 130, 635 neonates were screened from the year of 2013 to 2017. Neonates suspected for G6PD deficiency were further analyzed by quantitatively enzymatic assay and *G6PD* mutation analysis. The overall incidence of G6PD deficiency was 7.28%. A total of 14 *G6PD* mutations were identified, and different mutations lead to varying levels of G6PD enzymatic activities. The best cut-off value of G6PD activity in male subjects is 2.2 U/g Hb, same as conventional setting. In female population, however, the cut-off value is found to be 2.8 U/g Hb (sensitivity: 97.5%, specificity: 87.7%, AUC: 0.964) to best discriminate between normal and heterozygotes, and 1.6 U/g Hb (sensitivity: 82.2%, specificity: 85.9%, AUC: 0.871) between heterozygotes and deficient subjects. In conclusion, we have conducted a comprehensive newborn screening of G6PD deficiency in a large cohort of population from Guangxi, China, and first established a reliable cut-off value of G6PD activity to distinguish heterozygous females from either normal or deficient subjects.

## Introduction

Glucose-6-phosphate dehydrogenase (G6PD) deficiency is one of the most common monogenic diseases in human, involving about 400 million people worldwide^1,2^. G6PD deficiency is also known as favism (after intake of fava bean) because certain incentives such as beans or drugs can induce its occurrence. This disease, generally asymptomatic, is often manifested as acute hemolytic anemia and the resulting hyperbilirubinemia. Severe cases in neonatal period can develop into cerebral jaundice and thus lead to cerebral palsy with mental retardation^3–5^.

G6PD deficiency belongs to an X-linked recessive inborn error of metabolism that largely affects males (hemizygosity), whereas heterozygous females can be of normal, intermediate or deficient G6PD activity due to random chromosome X inactivation^1,6,7^. Routine newborn screening of G6PD deficiency applies one cut-off value (ranging from 2.10–2.60 U/gHb^8^) that could only discriminate between normal or G6PD enzymatic deficient subjects. A considerable proportion of heterozygous females with probable partial G6PD deficiency could be missed in clinical practice. Thus the cut-off values for neonatal screening of G6PD deficiency needs to be further differentiated and optimized between gender. In recent years, several groups have tried to set cut-off values of G6PD activity to discriminate among normal, heterozygous, and deficient individuals respectively^9,10^. However, the reported cut-off value varied in each study, probably due to the remarkable difference of study population and screening methods. Under such circumstances, a laboratory-specfic cut-off value needs to be established.

The *G6PD* gene, located in the long arm of chromosome X (Xq28), consists of 13 exons and 12 introns encoding 515 amino acids. Over 200 *G6PD* mutations have been reported worldwide, and the distribution of mutations is of racial and regional heterogeneity^11,12^. Up to now, the epidemiological data of neonatal G6PD deficiency in China remains elusive^13,14^. The relationship between different *G6PD* mutations and their phenotypes have not yet been fully established.

In this study, the results of G6PD screening and confirmative diagnosis in a total of 130,635 neonates, encountered at the Maternal and Child Health Hospital of Guangxi Zhuang Autonomous Region from January 2013 to March 2017, were retrospectively analyzed. The incidence of G6PD deficiency, mutation spectrum, genotype–phenotype correlation, and cut-off value settings for male and female neonatal screening in this region were explored.

## Results

### Analysis of G6PD activities

Among the 130,635 newborns (71511 males and 59124 females) screened for G6PD enzyme activity using fluorescent spot-test (FST), a total of 9583 cases (7832 males and 1751 females) were suspected as G6PD deficiency. The positive rate of initial screening was 7.34% (9583/130,635), 10.95% (7832/71511) and 2.96% (1751/59124) for the entire, male and female groups. And the G6PD enzyme activities (mean ± standard deviation) were 4.837 ± 1.603 U/g Hb, 4.742 ± 1.758 U/g Hb, and 4.952 ± 1.385 U/g Hb for the entire, male and female groups. Interestingly, the positive rate of G6PD deficiency presented with a decreased tendency, ranging from 7.55% (Year 2013), 7.50% (Year 2014), 7.37% (Year 2015), 6.91% (Year 2016), to 6.81% (Year 2017).

Our study cohort comprised of at least 29 ethnicities, including Zhuang, Han, Yao, Miao, Mulao, Tong, Man, Maonan, Tujia, Hui, Buyi, Yuenan, Jing and 16 other minorities. Among these 9583 neonates initially screened as G6PD deficiency, there were 5762 cases with information of ethnicity, including 3196 cases (55.47%) of Zhuang, 2316 cases (40.19%) of Han, 206 cases (3.58%) of Yao, 11 cases (0.19%) of Mulao, 10 cases (0.17%) of Miao and 9 cases (0.16%) of Tong ethnicity. The proportion of each other ethnicity accounts for less than 0.1%.

### Determination of *G6PD* mutations and G6PD/6PGD ratio

1566 (1229 males and 337 females) of 9583 neonates suspected with G6PD deficiency were further tested by both *G6PD* mutations analysis and quantitative G6PD enzymatic assay. It was found that 1553 cases (1221 males and 332 females) had either hemizygous, heterozygous, homozygous or compound heterozygous *G6PD* mutations (Table 1 and Supplementary Table 1), with a diagnosis rate of 99.17%. Based on that, the prevalence of G6PD deficiency in this population was estimated to be 7.28% (approximately equals to 7.34%* 99.17%).

**Table 1:** Allele frequency of different *G6PD* mutations in 1553 neonates.

Allele	rs_ID or HGMD_ID	Male hemizygotes	Female			Sum of alleles	Percentage (%)
			homozygotes	heterozygotes	Compound heterozygotes		
**c.1388 G** > **A (p.R463H)**	**rs72554664**	**445**	**13**	**66**	**62**	**599**	**35.4**
**c.1376 G** > **T (p.R459L)**	**rs72554665**	**328**	**18**	**52**	**49**	**465**	**27.5**
**c.95 A** > **G (p.H32R)**	**rs137852340**	**258**	**5**	**42**	**49**	**359**	**21.2**
**c.871 G** > **A (p.V291M)**	**rs137852327**	**78**	**0**	**13**	**13**	**104**	**6.1**
**c.1024 C** > **T (p.L342F)**	**rs137852342**	**64**	**2**	**10**	**17**	**95**	**5.6**
**c.392 G** > **T (p.G131V)**	**rs137852341**	**27**	**0**	**9**	**4**	**40**	**2.4**
**c.1004 C** > **A (p.A335D)**	**CM950506**	**7**	**0**	**1**	**6**	**14**	**0.8**
**c.196 T** > **A (p.F66I)**	**CM052878**	**4**	**1**	**0**	**0**	**6**	**0.4**
**c.1360 C** > **T (p.R454C)**	**rs398123546**	**3**	**0**	**0**	**0**	**3**	**0.2**
**c.835 A** > **T (p.T279S)**	**CM014189**	**2**	**0**	**0**	**0**	**2**	**0.1**
**C.592 C** > **T (p.R198C)**	**rs137852330**	**2**	**0**	**0**	**0**	**2**	**0.1**
**c.99 A** > **G (p.I33M)**	**CM950495**	**1**	**0**	**0**	**0**	**1**	**0.1**
**c.178 C** > **G (p.L60V)**	**NA**	**1**	**0**	**0**	**0**	**1**	**0.1**
**c.517 T** > **C (p.F173L)**	**rs137852343**	**1**	**0**	**0**	**0**	**1**	**0.1**
**Total**		**1221**	**39**	**193**	**200**	**1692**	**100**

A total of 14 *G6PD* mutations have been detected. All 14 mutations have been reported. Table 1 lists the frequency of each mutated allele. The allele frequency of c.1388 G > A (p.R463H) was the highest, accounting for 35.4% of all G6PD deficiency alleles. Follow by that, the alleles of c.1376 G > T (p.R459L), c.95 A > G (p.H32R), c.871 G > A (p.V291M), c.1024 C > T (p.L342F) and c.392 G > T (p.G131V) consist of 27.5%, 21.2%, 6.1%, 5.6% and 2.4%, respectively.

Among the 1553 cases carrying *G6PD* mutations, there were 606 cases with ethnicity recorded, including 302 cases of Zhuang (51.49%), 278 cases of Han (45.87%), 9 cases of Yao (1.49%) and 7 cases of other ethnicites (1.15%). We compared the frequency of different G6PD mutated alleles between Zhuang and Han ethnicity, and did not observe any significant variations as shown in Supplementary Table 2.

Among 332 female neonates, 139 cases carried either homozygous or compound heterozygous mutations, whereas the remaining 193 cases had a heterozygous mutation. The mean value of G6PD/6PGD ratio is 0.866 ± 0.372 in these 193 female carriers, among which 129 cases were confirmed as deficient by quantitative G6PD enzymatic assay (with a mean ratio of 0.693 ± 0.267) and 64 cases as non-deficient with a mean ratio of 1.269 ± 0.258.

### Correlation analysis of *G6PD* mutations and G6PD enzymatic activity

**Figure 1:**
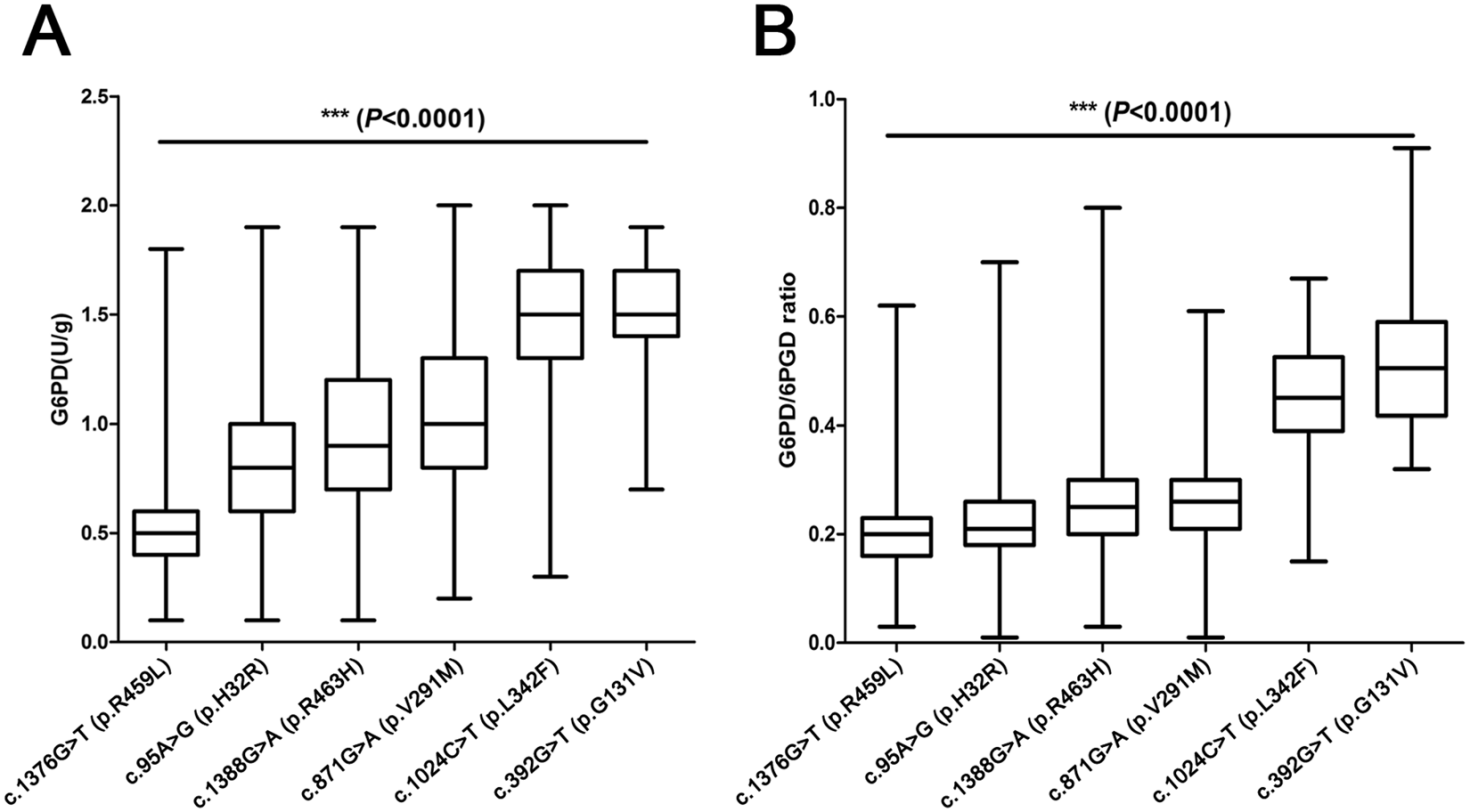
Correlation analysis of *G6PD* mutations and G6PD enzymatic activity. Different *G6PD* mutations had various levels of G6PD enzymatic activities, including (**A**) G6PD activity and (**B**) G6PD/6PGD ratio.

Six prevalent *G6PD* mutations were selected for correlation analysis with both G6PD activity and G6PD/6PGD ratio. It was found that different gene mutations had various levels of G6PD enzymatic activities (Fig. 1 and Table 2). The second most common allele of c.1376 G > T (p.R459L) had a largest decrease of enzymatic activity, followed by c.95 A > G (p.H32R), c.1388 G > A (p.R463H), c.871 G > A (p.V291M), c.1024 C > T (p.L342F) and c.392 G > T (p.G131V).

**Table 2:** Comparison of G6PD activity and G6PD/6PGD ratio among six prevalent gene mutations.

Genotype	Classification	G6PD activity	*P* value^*^					G6PD/6PGD ratio	*P* value				
c.1376 G > T	II	0.563 ± 0.265	<0.0001					0.197 ± 0.083	<0.0001				
c.95 A > G	II	0.832 ± 0.312		<0.0001				0.220 ± 0.084		<0.0001			
c.1388 G > A	II	0.969 ± 0.340			0.0020			0.259 ± 0.098			0.2140		
c.871 G > A	II	1.089 ± 0.402				<0.0001		0.259 ± 0.091				<0.0001	
c.1024 C > T	III	1.465 ± 0.334					0.3583	0.454 ± 0.108					0.1800
c.392 G > T	III	1.526 ± 0.296						0.517 ± 0.136					

^*^The value in each box represents the significance of difference between the two neighbouring *G6PD* mutations. P value less than 0.0001 indicates that there is significant difference of enzyme activity or G6PD/6PGD ratio among these six *G6PD* mutations.

### Cutoff value determination of G6PD activity

A total of 2789 neonates (1971 males and 818 females) were analyzed, including 2088 cases with *G6PD* mutations (1633 male hemizygotes, 135 female homozygotes or compound heterozygotes, 320 female heterozygotes) and 701 cases with no known *G6PD* mutations (338 males and 363 females). Among these neonates, 1932 cases were defined as suspected G6PD deficiency with decreased G6PD value (less than 2.20 UL/g Hb) at initial screening and 857 cases were defined as normal.

**Figure 2:**
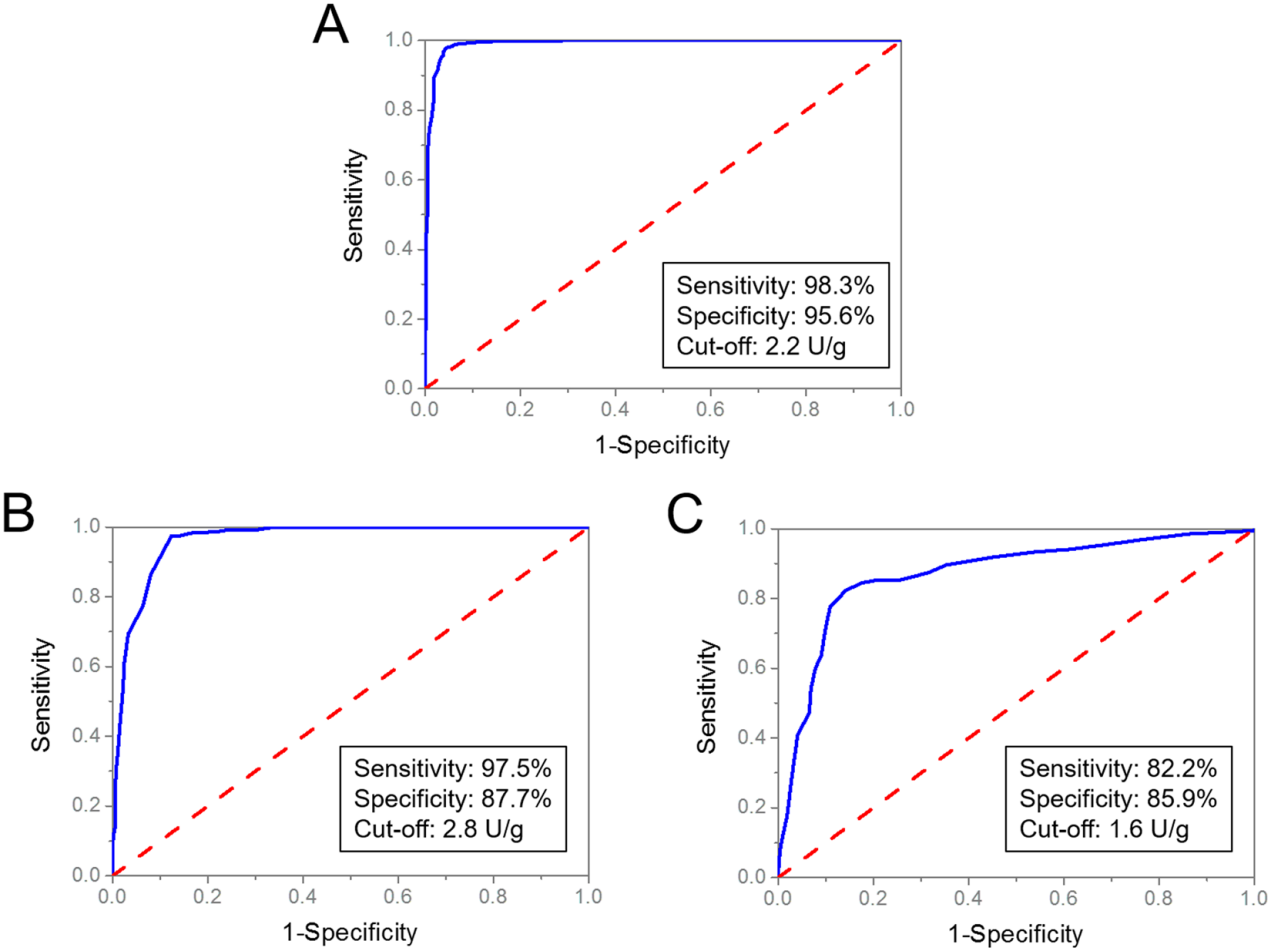
ROC curve analysis and determination of the cutoff value of G6PD activity in male and female groups separately, to discriminate between (**A**) normal vs hemizygous males, (**B**) normal vs heterozygous females, (**C**) heterozygous vs homozygous/compound heterozygous females. The curve is generated by plotting the true positive rate (sensitivity) against the false positive rate (1 − specificity). The accuracy was shown as the area under the ROC curve with 95% confidence interval. The optimal cut-off value was defined as one with the highest Youden’s index (=sensitivity + specificity − 1).

Receiver operating characteristics (ROC) curve analysis was performed to determine the cut-off values for male and female population in separate (Fig. 2). For male neonates, the cut-off value of G6PD activity between normal and G6PD deficient hemizygotes was 2.2 U/g Hb, which yielded a sensitivity of 98.3% and a specificity of 95.6% with the area under curve (AUC) at 0.988. For female neonates, the cut-off value between normal and female heterozygotes was 2.8 U/g Hb, which yielded a sensitivity of 97.5% and a specificity of 87.7% with AUC at 0.964. Meanwhile, the cut-off value between female heterozygotes and homozygotes/compound heterozygotes was 1.6 U/g Hb, which yielded a sensitivity of 82.2% and a specificity of 85.9% with AUC at 0.871.

## Discussion

G6PD deficiency is a major risk factor for the development of severe hyperbilirubinemia and increases the risk of bilirubin neurotoxicity. The prevalence and mutation spectrum could be various in different countries and different ethnic groups within a country. Guangxi Zhuang Autonomous Region is dominated with Zhuang minority, a distinct ethnic group from Han. In the present study, the frequency of G6PD deficiency among neonates is close to 7.28%. This incidence is higher than that reported in Guizhou (1.94%)^15^, Guangzhou (3.7%)^16^, Chaozhou (2.68%)^17^ and Jiangxi (3.6%)^18^ in southern China, whereas similar to that estimated in Greek (7.7%)^19^ and Indian (7.8%)^20^. This high prevalence could be related to the study objects of different ethnicity and the confirmative diagnosis methods applied. Also, cases having either hemizygous, heterozygous, homozygous or compound heterozygous *G6PD* mutations were defined as G6PD deficient subjects, considering female heterozygotes may also be deficient due to random inactivation of X chromosome.

G6PD deficiency is remarkable for its genetic diversity, different gene mutations cause different levels of enzyme deficiency and disease manifestation^21^. The data showed that c.1388 G > A, c.1376 G > T and c.95 A > G were the three most common *G6PD* mutations in this study cohort, accounting for 84% of total disease alleles, similar to other regions in south China^15,17,22^. All six common mutations found in these study belong to class II or III^23,24^. The enzyme activity of neonates with class II mutations were much lower than those with class III mutations, and even in the same class II, different mutations may cause different degree of enzyme deficiency with statistical significance (Table 2).

The *G6PD* gene is located on the X chromosome, thus in males it occurs only as a normal or deficient hemizygous genotype; but in females who have two copies of the X chromosome, one of which is randomly inactivated early in embryogenesis during the process of lyonization, it occurs to be more complex^25^. Homozygous females will be either normal or deficient for G6PD depending on the type of allele they possess. However, heterozygous females will be mosaic and have two erythrocyte populations consisting of normal and deficient cells. The proportions of these two types of red cell populations can be variable giving a diagnostic challenge on female heterozygotes^26^. In this study, we found 129 heterozygous females with abnormal Nitroblue tetrazolium (NBT) ratio, and 64 heterozygous females with normal NBT ratio. The rate of deficient to normal is estimated to be 2:1.

Several qualitative methods for G6PD deficiency screening are now available, but it has been shown that these methods have failed to detect partial deficient cases^27,28^. Considering the shortcomings of these screening methods, a few studies have been conducted with the purpose of setting cut-off values of G6PD activity, to discriminate among normal, heterozygous, and deficient individuals. But none of them can distinguish the heterozygous females from either normal or deficient subjects^9,10^. In this study, we focused on establishing cut-off values for G6PD screening based on a large cohort of subjects to increase the sensitivity in detecting female heterozygotes. First, the cut-off value (2.2 U/g Hb) in male subjects is same as conventional cut-off value, which is able to distinguish between normal and deficient subjects. Second, for female population, we first established a reliable cut-off value of G6PD activity to distinguish heterozygous females from either normal (2.8 U/g Hb) or deficient (1.6 U/g Hb) subjects. 43.75% (140 in 320) of heterozygous females with G6PD activities range from 2.2 to 2.8 U/g Hb could be missed if routine cut-off value of 2.2 U/g Hb was applied.

In conclusion, we conducted a comprehensive newborn screening of G6PD deficiency in a large cohort of population from Guangxi Province. The incidence and mutation spectrum of G6PD deficiency were elucidated, which could be useful for genetic counseling and prevention of this disease in Guangxi. Moreover, we first established a reliable cut-off value of G6PD activity to distinguish heterozygous females from either normal or deficient subjects. The cut-offs we have defined present the advantages of faster turnaround time and cost savings without additional genetic analysis.

## Methods

### Study design

A total of 130,635 newborns, encountered at the Newborn Screening Center of Guangxi, the Maternal and Child Health Hospital of Guangxi Zhuang Autonomous Region in China, were screened for G6PD deficiency from January 2013 to March 2017. This Center, as the first and largest newbron screening center in Guangxi, is in charge of the neonatal screening for about half of the total population in this area, and the coverage for newborn screening is 95.2% for this part of the country. The screening follows a standard screening protocol: a heel capillary blood sample was collected from the newborns between the 3rd and 5th day of life, adsorbed on a filter paper (S&S 903) and delivered to our neonatal screening laboratory where the modified fluorescent spot-test (FST) was applied to detect G6PD enzyme activity using the neonatal G6PD Kit (PerkinElmer, Wallac Oy, Turku, Finland). Subjects with decreased G6PD value (less than 2.20 UL/g Hb) were contacted and re-evaluated by quantitative G6PD enzymatic assay and/or *G6PD* mutation analysis.

For the quantitative evaluation of G6PD activity, the improved G6PD Nitroblue tetrazolium (NBT) Quantification Ratio Kit (Micky, Guangzhou, China) was used. Those with G6PD/6PGD ratio of ≤1.0 were considered as G6PD deficient^14,15^. For the detection of *G6PD* mutations, genomic DNA was extracted from the blood samples using TIANamp Blood DNA Kit (TIANGEN, Beijing, China). PCR was performed in a final reaction volume of 20.0 μL containing 2.0 μL of 10x PCR buffer (with 15.0 mM MgCl_2_), 0.5 μL MgCl_2_ (25 mM), 4.0 μL Q-solution (Qiagen), 3.2 μL dNTP mixture (2.5 mM), 0.4 μL forward (10 mM) and 0.4 μL reverse primers (10 mM), 0.2 μL of HotStarTaq DNA polymerase (Qiagen) and 200.0 ng of genomic DNA, total volume was made to 20.0 μL with nuclease-free water. PCR primer sequences were designed referring to the human GenBank, and 10 pairs of primers sequences (as shown in Supplementary Table 2) were used for amplication and sequencing of G6PD exon 2 to 13. Chromas software was applied for sequence analysis, and NCBI BLAST was used for DNA sequence alignment. This study was approved by the Medical Ethics Committee of Guangxi Maternal and Child Health Hospital. Informed consent was obtained from the parents of the patients. All methods were performed in accordance with the approved guidelines (http://www.nature.com/srep/policies/index.html#experimental-subjects).

### Statistical Analysis

The data were analyzed using SPSS version 19.0 (SPSS, Chicago, IL). T-test was used to evaluate the mean values of G6PD activity and G6PD/6PGD ratio among different *G6PD* genotypes. For the comparison of multiple means, Kruskal-Wallis test was used. P value less than  0.05 was considered as statistically significant. Receiver operating characteristics (ROC) curve analysis was performed using OriginPro 9.1 software (OriginLab Corp., Northampton, MA, USA), to determine the best cut-off value and to evaluate the performance of diagnosis at different levels of G6PD activity. Youden index (YI) was calculated as (specificity + sensitivity) − 1.

## Electronic supplementary material

Supplementary Materials

## References

[1] Galatas B (2017). Heterogeneity of G6PD deficiency prevalence in Mozambique: a school-based cross-sectional survey in three different regions. Malaria journal.

[2] Valencia SH, Ocampo ID, Arce-Plata MI, Recht J, Arevalo-Herrera M (2016). Glucose-6-phosphate dehydrogenase deficiency prevalence and genetic variants in malaria endemic areas of Colombia. Malaria journal.

[3] Ogunlesi TA, Dedeke IO, Adekanmbi AF, Fetuga MB, Ogunfowora OB (2007). The incidence and outcome of bilirubin encephalopathy in Nigeria: a bi-centre study. Nigerian journal of medicine: journal of the National Association of Resident Doctors of Nigeria.

[4] Olusanya BO, Osibanjo FB, Mabogunje CA, Slusher TM, Olowe SA (2016). The burden and management of neonatal jaundice in Nigeria: A scoping review of the literature. Nigerian journal of clinical practice.

[5] Cunningham AD, Hwang S, Mochly-Rosen D (2016). Glucose-6-Phosphate Dehydrogenase Deficiency and the Need for a Novel Treatment to Prevent Kernicterus. Clinics in perinatology.

[6] Boonyuen U (2016). Detailed functional analysis of two clinical glucose-6-phosphate dehydrogenase (G6PD) variants, G6PDViangchan and G6PDViangchan + Mahidol: Decreased stability and catalytic efficiency contribute to the clinical phenotype. Molecular genetics and metabolism.

[7] Mbanefo EC (2017). Association of glucose-6-phosphate dehydrogenase deficiency and malaria: a systematic review and meta-analysis. Scientific reports.

[8] Newborn Screening Group, B. D. P. a. C. S. C. Chinese Preventive Medicine Society; Clinical Biochemical Genetics Specialized Committee, Medical Genetic Branch, Chinese Medical Association; Clinical Genetics Group, Adolescent Medical Specialized Committee, Chinese Medical Association. Expert consensus on the newborn screening, diagnosis and treatment of glucose −6- phosphate dehydrogenase deficiency. *Chinese Journal of Pediatrics* **6**, 411–414 (2017).

[9] Miao JK (2013). Determination of optimal cutoff value to accurately identify glucose-6-phosphate dehydrogenase-deficient heterozygous female neonates. Clinica chimica acta; international journal of clinical chemistry.

[10] Laouini N (2017). Determination of glucose-6-phosphate dehydrogenase cut-off values in a Tunisian population. Clinical chemistry and laboratory medicine.

[11] Jamwal M (2017). Next-generation sequencing unravels homozygous mutation in glucose-6-phosphate isomerase, GPIc.1040G > A (p.Arg347His) causing hemolysis in an Indian infant. Clinica chimica acta; international journal of clinical chemistry.

[12] Doss CG (2016). Genetic Epidemiology of Glucose-6-Dehydrogenase Deficiency in the ArabWorld. Scientific reports.

[13] Zhong DN, Gao ZY, Liu YN, Liu Y, Wei LM (2009). [Relationship between glucose-6-phosphate dehydrogenase gene mutations and neonatal jaundice in Naning, Guangxi]. Zhongguo dang dai er ke za zhi = Chinese journal of contemporary pediatrics.

[14] Yan T (2006). Incidence and complete molecular characterization of glucose-6-phosphate dehydrogenase deficiency in the Guangxi Zhuang autonomous region of southern China: description of four novel mutations. Haematologica.

[15] Huang S (2016). Molecular newborn screening of four genetic diseases in Guizhou Province of South China. Gene.

[16] Jiang J (2014). Screening and prevention of neonatal glucose 6-phosphate dehydrogenase deficiency in Guangzhou, China. Genetics and molecular research: GMR.

[17] Yang H (2015). Incidence and molecular characterization of Glucose-6-Phosphate Dehydrogenase deficiency among neonates for newborn screening in Chaozhou, China. International journal of laboratory hematology.

[18] Hu R (2015). Molecular epidemiological investigation of G6PD deficiency by a gene chip among Chinese Hakka of southern Jiangxi province. International journal of clinical and experimental pathology.

[19] Molou E (2014). Glucose-6-Phosphate Dehydrogenase (G6PD) deficiency in Greek newborns: the Mediterranean C563T mutation screening. Scandinavian journal of clinical and laboratory investigation.

[20] Ramadevi R, Savithri HS, Devi AR, Bittles AH, Rao NA (1994). An unusual distribution of glucose-6-phosphate dehydrogenase deficiency of south Indian newborn population. Indian journal of biochemistry & biophysics.

[21] Lee J (2017). Genetic Profiles of Korean Patients With Glucose-6-Phosphate Dehydrogenase Deficiency. Annals of laboratory medicine.

[22] Peng Q (2015). Large cohort screening of G6PD deficiency and the mutational spectrum in the Dongguan District in Southern China. PloS one.

[23] Jiang W (2006). Structure and function of glucose-6-phosphate dehydrogenase-deficient variants in Chinese population. Human genetics.

[24] Glucose-6-phosphate dehydrogenase deficiency. WHO Working Group. *Bulletin of the World Health Organization* **67**, 601–611 (1989). PMC2491315 2633878

[25] van den Broek L, Heylen E, van den Akker M (2016). Glucose-6-phosphate dehydrogenase deficiency: not exclusively in males. Clinical case reports.

[26] Gunawardena S (2017). Prevalence of G6PD deficiency in selected populations from two previously high malaria endemic areas of Sri Lanka. PloS one.

[27] Keihanian F (2017). Comparison of quantitative and qualitative tests for glucose-6-phosphate dehydrogenase deficiency in the neonatal period. International journal of laboratory hematology.

[28] Kaplan M, Hammerman C (2011). Neonatal screening for glucose-6-phosphate dehydrogenase deficiency: biochemical versus genetic technologies. Seminars in perinatology.

