# Supplementary material for: Newborn screening of glucose-6-phosphate dehydrogenase deficiency in Guangxi, China: determination of optimal cutoff value to identify heterozygous female neonates
Source: Sci Rep. 2018 Jan 16;8:833. doi: 10.1038/s41598-017-17667-6 (PMC5770456; doi:10.1038/s41598-017-17667-6)
Supplement: Supplementary file 1 — Supplementary Materials [file 41598_2017_17667_MOESM1_ESM.doc]

**Supplementary Materials for**

**Newborn screening of glucose-6-phosphate dehydrogenase deficiency in Guangxi, China: determination of optimal cutoff value to identify heterozygous female neonates**

**Chunyun Fu^1, 2†^, Shiyu Luo^1, 2†^, Qifei Li^3†^, Bobo Xie^1, 2^, Qi Yang^1, 2^, Guoxing Geng^1, 2^, Caijuan Lin^1, 2^, Jiasun Su^1, 2^, Yue Zhang^1, 2^, Jin Wang^1, 2^, Zailong Qin^1, 2^, Jingsi Luo^1, 2^, Shaoke Chen^2, 4*^, Xin Fan^1, 2*^**

**This file includes:**

**Supplementary Table 1-3**

**Supplementary Table 1. Summary of *G6PD* genotype in 1553 neonates.**

| **Genotype** | **No. of males** | **No. of females** | **Genotype** | **No. of males** | **No. of females** |
| --- | --- | --- | --- | --- | --- |
| c.95A>G (p.H32R) (hem/hom) | 258 | 5 | c.871G>A (p.V291M) (het) | - | 13 |
| c.95A>G (p.H32R) (het) | - | 42 | c.871G>A (p.V291M) (het)/ c.1376G>T (p.R459L) (het) | - | 6 |
| c.95A>G (p.H32R) (het)/ c.1376G>T (p.R459L) (het) | - | 14 | c.871G>A (p.V291M) (het)/c.1388G>A (p.R463H) (het) | - | 1 |
| c.95A>G (p.H32R) (het)/c.1004C>A (p.A335D) (het) | - | 2 | c.1004C>A (p.A335D) (hem) | 7 | - |
| c.95A>G (p.H32R) (het)/c.1024C>T (p.L342F) (het) | - | 4 | c.1004C>A (p.A335D) (het) | - | 1 |
| c.95A>G (p.H32R) (het)/c.1388G>A (p.R463H) (het) | - | 23 | c.1024C>T (p.L342F) (hem/hom) | 64 | 2 |
| c.95A>G (p.H32R) (het)/c.871G>A (p.V291M) (het) | - | 6 | c.1024C>T (p.L342F) (het) | - | 10 |
| c.99A>G (p.I33M) (hem) | 1 | - | c.1024C>T (p.L342F) (het)/c.1376G>T (p.R459L) (het) | - | 4 |
| c.178C>G (p.L60V) (hem) | 1 | - | c.1024C>T (p.L342F) (het)/c.1388G>A (p.R463H) (het) | - | 9 |
| c.196T>A (p.F66I) (hem/hom) | 4 | 1 | c.1360C>T (p.R454C) (hem) | 3 | - |
| c.392G>T (p.G131V) (hem) | 27 | - | c.1376G>T (p.R459L) (hem/hom) | 328 | 18 |
| c.392G>T (p.G131V) (het) | - | 9 | c.1376G>T (p.R459L) (het) | - | 52 |
| c.392G>T (p.G131V) (het)/c.1388G>A (p.R463H) (het) | - | 2 | c.1376G>T (p.R459L) (het) /c.392G>T (p.G131V) (het) | - | 2 |
| c.517T>C (p.F173L) (hem) | 1 | - | c.1376G>T (p.R459L) (het)/c.1388G>A (p.R463H) (het) | - | 23 |
| c.592C>T (p.R198C) (hem) | 2 | - | c.1388G>A (p.R463H) (hem/hom) | 445 | 13 |
| c.835A>T (p.T279S) (hem) | 2 | - | c.1388G>A (p.R463H) (het) | - | 66 |
| c.871G>A (p.V291M) (hem) | 78 | - | c.1388G>A (p.R463H) (het)/c.1004C>A (p.A335D) (het) | - | 4 |

hem: hemizygous, hom: homozygous, het: heterozygous

**Supplementary Table 2. Frequency of different *G6PD* mutated alleles between Zhuang and Han ethnicity**

| **Allele** | **Frequency per ethnicity*** | |
| --- | --- | --- |
|  | **Zhuang** | **Han** |
| **c.1388G>A(p.R463H)** | 36.66% (125) | 35.23% (99) |
| **c.1376G>T(p.R459L)** | 26.69% (91) | 28.83% (81) |
| **c.95A>G(p.H32R)** | 21.99% (75) | 19.22% (54) |
| **c.871G>A(p.V291M)** | 4.99% (17) | 6.41% (18) |
| **c.1024C>T(p.L342F)** | 6.16% (21) | 6.76% (19) |
| **c.392G>T(p.G131V)** | 2.64% (9) | 2.85% (8) |
| **c.1004C>A(p.A335D)** | 0.88% (3) | 0.71% (2) |
| **c.196T>A(p.F66I)** | 0.00% (0) | 0.36% (1) |

***** present in the format of percentage (No. of alleles)

**Supplementary Table 3. *G6PD* primer sequence for amplification and sequencing**

| **Exon** | **Forward primer (5’→3’)** | **Reverse primer (5’→3’)** | **Product length (bp)** |
| --- | --- | --- | --- |
| **2** | TCTAACCCATCAACCACTCC | AGGCACTTCCTGGCTTTT | 336 |
| **3-4** | CAGCCACTTCTAACCAC | AACCAGGCTGGGGGAG | 308 |
| **5** | TGTCTGTGCTGCCTGCTTT | AAAGGCGGTGTTTCGTGGA | 614 |
| **6** | AGGTGTTGAGCCAGAGGGTC | GAGGCAGTGGGCCAGGTG | 398 |
| **7** | GGCAAGGAGATGGTGCAGAA | TTCACCTGGTTCAAGGGCAT | 659 |
| **8** | AAGACAAGGGGGATCAGGAA | TGTGCTCAGAGGTGGTGACTT | 365 |
| **9** | TATGTGACCAGGGAAGGCCA | CCTCAGCACCAGCTCTCTCA | 555 |
| **10** | GGAGCTCCCACTGAGACACT | TGCTGATGCCACTGCCTG | 399 |
| **11-12** | GCAGTGGCATCAGCAAGA | AGTGACGGGTGGAGGAGA | 401 |
| **13** | TATGGCAGGTGAGGAAAGG | AGGTCAATGGTCCCGGAGT | 285 |
